# Interplay of trauma-triggered auto-inflammation and T-cell auto-reactivity in posttraumatic dystrophy

**DOI:** 10.3389/fimmu.2025.1404161

**Published:** 2025-06-18

**Authors:** Egmont Scola

**Affiliations:** Department of Traumatology, Dietrich-Bonhoeffer-Klinikum Neubrandenburg, affiliated Hospital of Medical School of University Greifswald, Mecklenburg-Vorpommern, Germany

**Keywords:** damage-associated molecular pattern, innate immunity, autoreactivity, NKT cell, AV-shunt, hyperperfusion, hypoxia, posttraumatic dystrophy

## Abstract

Damage-associated molecular patterns (DAMPs) cause sterile auto- inflammation after a traumatic incident in human tissues via innate immunity. The auto-reactivity of natural killer T cells (NK-T cells) instigated by DAMP-activated exocytosis of dendritic cell (DC) vesicles forces implementation of T helper cells, which aggravate inflammatory reactions such as AV shunts and hyperperfusion of the ROI with hypoxia of capillaries. For example, in trauma patients, elevated venous pO_2_ was found compared to that in the contralateral extremity of > 20 mmHg 2.66 kPa. Scintigraphic perfusion of the ROI showed elevated values of over 90% on average compared with the healthy side. These findings suggest local capillary stasis, hypoxia, and acidosis. In the initial process, macrophages and dendritic cells play an important role, along with DAMPs, in the activation of innate immunity. Additional tissue-homing NKT cells are activated by releasing pro-inflammatory cytokines. The resulting “cytokine storm” opens options for perpetuation by diverse autocrine loops and inflammasomes. Finally, antibodies against self-molecules are directed against cells and tissues. In a biological sense, this represents the worst scenario in chronic-aseptic inflammatory reactions after trauma and must be fought from the beginning to avoid chronification and spreading, which can lead to fibrosis and functional impairment of the injured extremity. This is the feared endpoint of posttraumatic dystrophy.

## 1 Introduction

Since Sudeck in 1901 (1), trauma surgery has recognized posttraumatic dystrophy (PTD) as a condition that develops after traumatic damage to cells and tissues in the peripheral extremities, usually near small or large joints. The clinical symptoms were the same as those described by Celsus (25 BC–50 AD) and Galen (129–199 AD): *rubor* (redness), *tumor* (swelling), *calor* (heat), *dolor* (pain), and *functio laesa* (malfunction).

In the second half of the 19^th^ century, with the discovery of microbes, the distinction between microbial or septic inflammation and nonmicrobial or aseptic inflammatory reactions became apparent. In 1888, killed vaccines and the first antigen and antibody reactions were discovered, leading Talmage to believe that immunology was born that year (2). It took another century for Janeway Jr. 1989 (3) to write his seminal work, “Approaching the Asymtote? Evolution and Revolution in Immunology”, which introduced the concepts of “pattern recognition receptors” (PRRs) and “pathogen-associated molecular patterns” (PAMPs). Building on this concept, Matzinger (4, 5) and Seong (6) developed the term “Damage-associated molecular patterns” (DAMPs) in 1994, 2002, and 2004, respectively, to explain the source of aseptic inflammation. However, when primarily posttraumatic auto-inflammation fails to resolve, chronification occurs, resulting in fibrosis of the soft tissues and loss of function of the affected limb (7).

The dominant symptom is pain, which normally disappears during the healing period after incidental trauma. Sometimes, the pain worsens intensively and is resistant to therapeutic approaches. The reason was assumed in the sympathetic nervous system, so the descriptive diagnosis “reflex sympathetic dystrophy (RSD)” was used for a long period (8) (see **Box 1**). In cases of RSD, not all patients appeared to have sympathetically maintained pain, nor were all dystrophic. This observation was made by other medical departments too, which also used the diagnosis of non-traumatic diseases with chronic pain sensations of unknown etiologies. Therefore, the task force on taxonomy of the International Association for Study of Pain (IASP) proposed in a special consensus conference 1994 in Orlando/FLA that the terminus “Complex regional pain syndrome, type I” (CRPS I) should be used in these morbid developments instead of RSD. Since then, PTD in trauma patients has been termed CRPS I (13, 16).

Box 1 **Posttraumatic Dystrophy PTD - an Odyssey.**

To honor Prof. Dr. Paul H. M. S. Sudeck after his death in 1945, ***F. Oehlecker in 1948*** (9) proposed naming Posttraumatic Dystrophy after its first describer and clinical researcher, P. Sudeck (in 1901). This recommendation was accredited by the surgical community, and from then on Posttraumatic Dystrophy was referred to as ***“Morbus Sudeck”*** in Europe. The etiology and causal therapy remained unknown.

In the United States, ***J. A. Evans in 1946*** (8) saw posttraumatic inflammation as a sympathetic reflex of the vessels and termed this complication as ***“Reflex sympathetic dystrophy, RSD.”*** This diagnosis became prevalent in the Anglo-American region, where the therapy was unspecific and primarily involving analgesic procedures. ***C. Blumensaat in 1956*** (10) wrote the first review titled “The present extent of knowledge of the Sudeck syndrome” [Der heutige Stand vom Sudeck Syndrom], in which elevated venous pO2 values and AV shunts in the affected limb were mentioned for the first time in this context. These findings were not further discussed, and the management of M. Sudeck remained unclear.

In cases of RSD, it was observed that not all patients experienced sympathetically maintained pain, nor were all cases dystrophic. This observation was also made by other medical departments, which used the RSD diagnosis for non-traumatic diseases with chronic pain of unknown etiologies. Consequently, the task force on taxonomy of the ***International Association for Study of Pain (IASP)*** proposed in a special ***consensus conference 1994*** in Orlando/FLA that the terminus “***Complex regional pain syndrome, type I*** *”* ***(CRPS I)*** should be used in these morbid developments instead of RSD (11). Since then, Sudeck syndrome in trauma patients has been termed CRPS I.

**T. J. Coderre in 2011** (12) made an update in CRPS I and claimed a still lacking etiology for this posttraumatic complication. **R. N. Harden in 2013** (13) wrote a comprehensive update about “Complex regional pain syndrome: Practical Diagnostic and Treatment Guidelines, 4^th^ edition.” He assumed a disturbed microcirculation by multiple factors without reference to the etiology. **F. Birklein 2017** (14) proposed a medical treatment in the initial phase of Posttraumatic Dystrophy with glucocorticoids based on his own good experiences but without reflection on evidence-based theory.

Most recently, **T. Okumo in 2022** (15) wrote in his conclusions, ”Even with the accumulation of clinical research over the decades, the **etiology and pathogenesis of CRPS**, as well as appropriate treatment strategies, **are still being explored.**”

However, Posttraumatic Dystrophy occurs worldwide after traumatic incidents and is predominantly seen by trauma surgeons. Diagnostic and therapy of PTD should belong to their skills to recognize this fateful complication after injuries instantly. The therapy must begin as soon as possible without any retardation. The here presented work sheds light on the lacking etiology, which needs further research to prove the reliability of recommendations for the therapy.

(The huge number of other terms and classifications for “PTD” are not mentioned.)

The main features of CRPS are mild trauma, such as a fracture or soft tissue lesion. As a diagnostic criterion, “this diagnosis is excluded by the existence of conditions that would otherwise account for the degree of pain and dysfunction” (13, 16).

The point of view in traumatology is that most trauma patients are affected by healthy conditions, except for trauma and posttraumatic complications. Nevertheless, even several attempts at classification by clinical symptoms cannot explain the etiology of CRPS I or provide evidence-based therapy (13).

In the early stage after trauma, our clinical examinations revealed an elevated concentration of vpO_2_ in the affected limb compared with the healthy contra lateral limb (17–19). If this difference persisted after 4 to 6 weeks (vpO_2_ > 20 mmHg [2.66 kPa]) and the symptoms of aseptic inflammatory reaction continued, disturbed microcirculation was assumed, with the risk of hypoxia, acidosis, and the formation of free radicals. The application of rheologically active hydroxyethyl starch relieved the symptoms and normalized venous oxygenation values. None of the patients showed rebound effects. However, when hydroxyethyl starch is withdrawn from the market due to side effects in intensive care unit (ICU) patients the question about the true origin of the PTD and its causal therapy remains.

This article reviews the traditional symptoms of posttraumatic inflammation, their interpretations, and the roles of humoral and cellular participation in innate immunity. The ability to augment and perpetuate inflammatory responses is demonstrated through an analysis of scientific literature.

## 2 Premise

The following subsections 2.1 to 2.10 present relevant facts from the scientific literature that support the effort to determine the etiology of PTD, which appears to be rooted in the immunological aspect of posttraumatic inflammation. Section 2.11 (clinical aspect) to 2.14 (clinical aspect) focus on clinical symptoms resulting from auto-inflammatory processes following traumatic impact.

### 2.1 Development of the auto-inflammatory reaction: an outline

Posttraumatic inflammatory reactions originate in innate immunity, which is present in all cells of the human body and is activated in response to endogenous and/or exogenous stimuli.

Activation is triggered by endogenous factors, such as cell detritus or locally disrupted homeostasis, which causes cellular stress. When signs of damage or danger occur, they immediately activate the innate immunity through various pathways. Therefore, these molecules are named damage (or danger)-associated molecular patterns (DAMPs). DAMPs are a heterogeneous group of molecules, most of which remain “incognito” as part of larger molecular complexes that constitute functional tissues and organs. Their primary role is to signal tissue damage or danger through previously unrecognized molecules. The purpose of DAMPs is to induce immediate tissue repair via innate immunity responses or to amplify primary inflammation if DAMP signals persist from any local source. While this process ensures a rapid and effective reaction, it is non-specific.

Exogenous stimuli, such as invading microbes or their pathogenic molecules, can spread throughout the body through networks of nerves, vessels, and blood, resulting in the symptoms of general illness. Defense against these pathogens is normally specific to lymphocytes, which are part of adaptive immunity. Pathogens and their molecules are known as PAMPs. The defense activity of adaptive immunity involves an incubation period due to the delayed production of antibodies, which must be bridged by innate immunity. Therefore, DAMPs and PAMPs are considered complementary systems. Before cellular defense begins, humoral defense of innate immunity initiates an intense inflammatory reaction in the damaged area. Several immune cells are polarized and activated in the tissue before inflammatory reactions occur (20, 21). This study focuses on aseptic innate auto-inflammation and DAMP activity.

### 2.2 Free radicals, ROS, and RNS

Posttraumatic hypoxia triggers the generation of free radicals. Hypoxia and inflammation are intertwined at the molecular, cellular, and microvascular levels (22). Free radical formation is facilitated by the presence of oxygen (ROS) or nitrogen (RNS) in acidic environments. Furthermore, under anaerobic conditions mitochondria produce free radicals (23). Mitochondrial ROS, along with pseudo-peroxidase cytochrome *c*, also releases free radicals that react with the mitochondrial phospholipid cardiolipin to produce an oxidized phospholipid (oxPL), a signal of programmed cell death.

**Figure 1:**
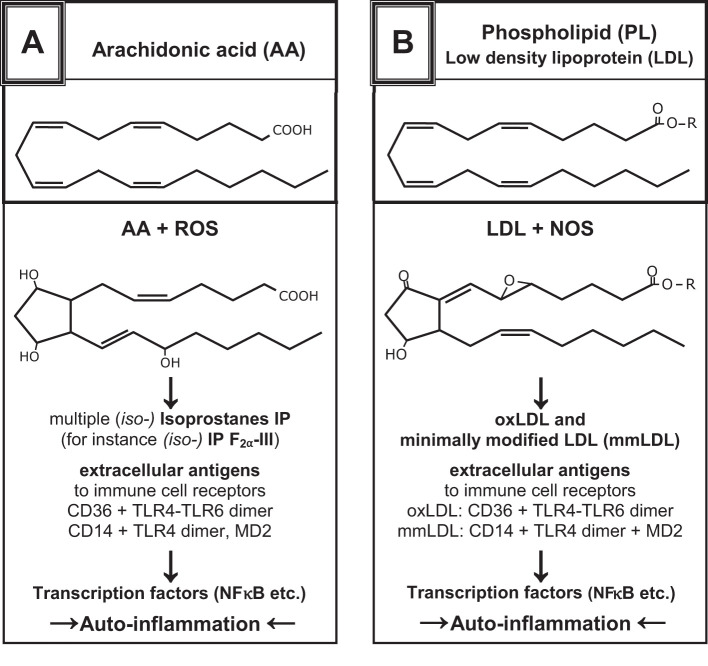
Examples show the chemical structures of non-enzymatically altered (self-) products from the oxidation of arachidonic acid via free radical-catalyzed peroxidation **(A)** and unsaturated phospholipids **(B)**, partially with high antigenic potential [mod. from (24–29)]. They can yield several oxidation-specific epitopes such as isoprostanes with varying ring structures and oxidized PL. The antigenic effects of oxPL provoke the development of natural antibodies by B1-cells (IgM), which enables the recognition of PRRs and activates innate immune responses (30).

Superoxides are responsible for modifying the molecular structure of membrane lipids (**Figure 1**) (22–24, 31–33). Fatty acids, which are organic substances and used in the construction of biological cell walls, share similarities with micro-organisms like viruses and bacteria. When fatty acids evolve in the phagolysosomes of phagocytic cells, the same compatibility control is performed as that for self-lipids. However, the phagocyte or T-cell cannot differentiate between human and microbial origins; therefore, the result will be the same. Polyunsaturated fatty acids (PUFAs) form antigenic oxPLs with free radicals, which act as self-ligands for receptors on inflammatory cells in innate immunity (25–30). If “unwanted” activation of T cells of innate immunity occurs, resulting in the development of chronic-aseptic inflammatory reactions caused by autogenic detritus, the term “T-cell autoreactivity” is used (see subsection 2.9).

These oxPLs are found intracellularly (cytosol) and extracellularly (interstice) where they can be identified by IgM auto-antibodies, soluble scavenger receptors, complement proteins, adapter proteins, PRRs, toll-like receptors (TLRs), RIG-like receptors (RLRs), and NOD-like receptors (NLRs) (21, 29, 31–33). In the macrophage membrane (pro-inflammatory, see subsection 2.6), stimulation with nicotine adenine dinucleotide phosphate (NADPH) oxidase (NOX2) produces superoxide anions (·O_2_˙). Superoxide dismutase (SOD) then transforms them into hydrogen peroxide (H_2_O_2_), which can be further decomposed into hydroxyl radicals (OH^–^/·OH) or by myeloperoxidase to hypochlorous acid HClO^-^. The cytotoxicity of ROS is enhanced by RNS like NO˙, nitrogen dioxide (NOO^-^), and peroxynitrite (ONOO^-^) (25–27, 34) (**Table 1**).

**Table 1:** Resolution of selected oxygen and nitrogen radicals, details see the text.

radical	adduct	enzyme	product
·O_2_^–^ superoxide	+ NO˙	–	ONOO^–^ peroxinitrite
ONOO^–^ peroxinitrite	–	+ iNOS	superoxide ·O_2_^–^(and peroxide O_2_^–^)
2 ·O_2_^–^ superoxide	+ 2 H^+^	superoxide dismutase (SOD)	H_2_O_2_hydrogen peroxide + O_2_
H_2_O_2_ hydrogen peroxide	+ chloride Cl^–^	(myelo-) peroxidase	HClO^–^ hypochlorus acid
2 H_2_O_2_ hydrogen peroxide	–	catalase	2 H_2_O + O_2_
H_2_O_2_ hydrogen peroxide	+ Fe^2+^	–	Fe^3+^ + OH^–^ + hydroxyl radical ·OH (Fenton reaction)

**Figure 2:**
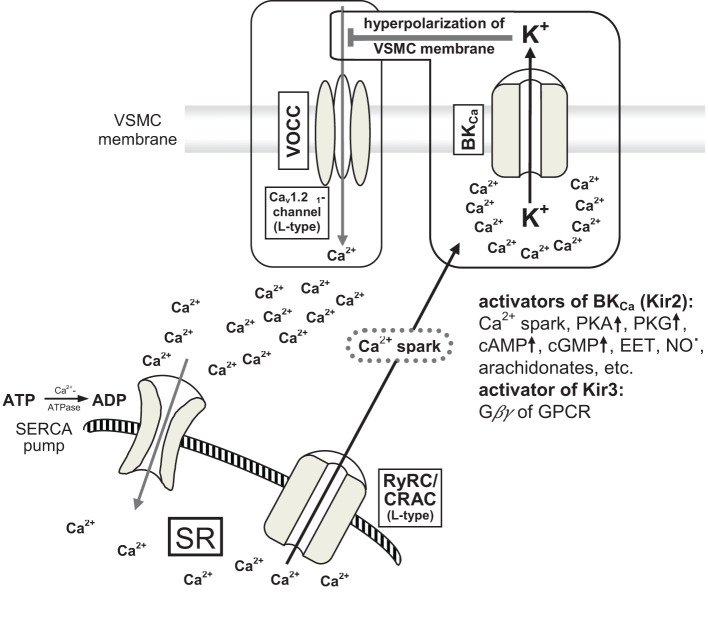
[mod. based on (36–39)]. Schematic presentation of big conductance calcium-activated potassium channel (BK_Ca_-channel) and interaction with large conductance voltage operated calcium channels (VOCCs) resulting in relaxation of VSMCs by hyperpolarization of cell membranes (~ 20 mV). VSMC, vascular smooth muscle cell; VOCC, large conductance voltage operated calcium channel (L-type Ca_v_1.2α_1_ calcium channel); BK_Ca_, big conductance calcium-activated potassium channel; calcium spark: local enhancement of calcium concentration up to 10–100 µM; PKA and PKG proteinkinases; cAMP and cGMP cyclic nucleotides; EET epoxy-eicosatrienoic acid (arachidonate); G*βγ* small G*β* and G*γ* effector proteins of GPCR (G protein-coupled receptor); Kir3 inwardly rectifying potassium channel (= BK_Ca_) type 3; RyRC, ryanodin receptor calcium channel; CRAC, calcium release-activated calcium channel (L-type); SR, sarcoplasmatic reticulum; SERCA, sarco-endoplasmatic reticulum calcium ATPase.

From the beginning, RNS such as nitric oxide (NO**˙**) are liberated from the endothelium of arteries (35). These are produced by physiological enzymes like endothelial nitric oxide synthase (eNOS) and neuronal nitric oxide synthase (nNOS). This short-lived gaseous transmitter diffuses freely through the cell walls and supports the relaxation of VSMCs via pathways involving cGMP, PKA, and myosin light chain phosphatase (MLCP). For instance, it activates Ca^2+^-dependent big potassium channels (BK_Ca_, inwardly rectifying potassium channels Kir2.1) (36) and subsequently blocks L-type voltage operated calcium channels (VOCCs) due to hyperpolarization of the VSMC membrane (**Figure 2**). This relaxation signal is transduced by connexins to other VSMCs of the single-unit type. Additionally, signals from endothelial cells (ECs) to VSMCs are transmitted through MEGJs, involving Cx40 (36–41).

A large amount of peroxynitrite can form when induced nitric oxide synthase (iNOS) uses oxygen from ROS like superoxide anions (·O_2_^·^), resulting in peroxynitrite instead of NO**˙** (42). While this potent cytotoxic species is intended to combat pathogens, it also inadvertently damages self-structure and is highly destructive. This occurrence is critical because iNOS, which is non-physiological and independent of Ca^2+^ and calmodulin, can produce up to 1.000 times more and longer-lasting peroxynitrite than the physiological eNOS or nNOS produce the free radical NO**˙** (43). The interaction of iNOS with peroxynitrite (ONOO^–^) or superoxide anions (·O_2_^⬝^) leads to further production of peroxynitrite and superoxide, along with a loss of heme molecule function in soluble guanylyl cyclase sGC enzyme. This interrupts the negative feedback to NO**˙** and cGMP, leading to massive production of cyclic guanosine monophosphate (cGMP), protein kinase G (PKG), and induced vasodilation (44).

These processes are pertinent for promoting normal wound healing and are also foundational for enhanced defense reactions when needed.

### 2.3 Redox-sensitive transcription factors

Shortly after a traumatic incident, redox-sensitive transcription factors are activated. These include nuclear factor of kappa-light-chain-enhancer in activated B-cells (NF-κB), hypoxia-inducible factor 1 (HIF1), nuclear factor E2-related factor 2 (Nrf2) and cAMP-responsive element-binding protein (CREB), which modulate transcription. These factors initiate the expression of target genes that regulate inflammation, cell growth, development, immunity, and apoptosis. Notably, NF-κB influences over 500 target genes (21, 45–47), HIF1 affects more than 500 throughout the genome (48), and Nrf2 binds to AREs among others (49). The primary objectives are to restore the microcirculation by alleviating hypoxia and promoting the phagocytosis of cell detritus. Achieving homeostasis is essential for definitive healing.

### 2.4 Oxidative burst

**Figure 3:**
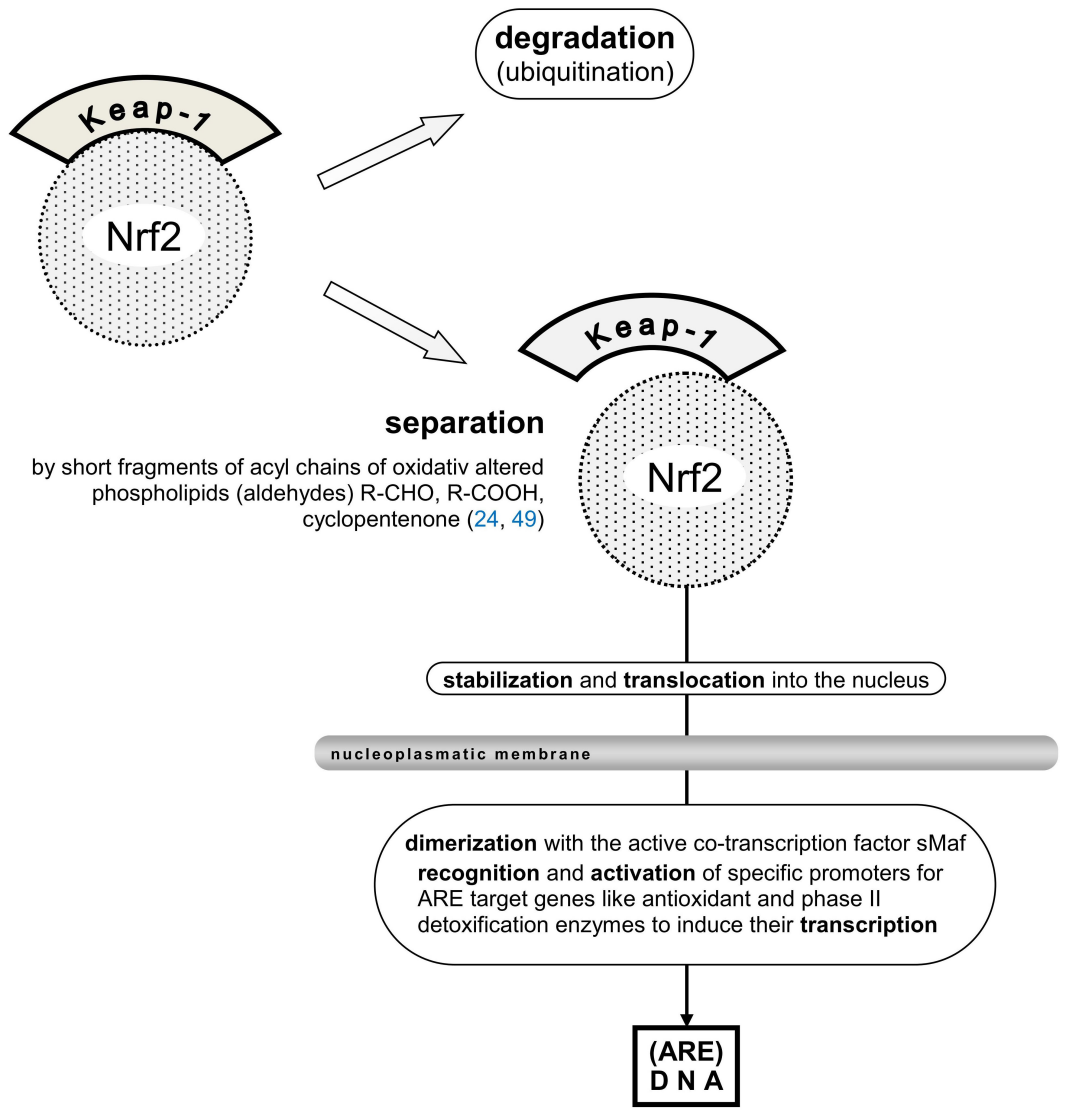
[mod. based on (24, 49)]. Schematic presentation of Nrf2 transcription factor inhibitor protein Keap-1, (partial) separation from the Nrf2 moiety, stabilization, and translocation into the nucleus are shown. Mandatory dimerization with the active co-transcription factor sMaf, recognition of specific ARE in the promoters of target genes like antioxidant and phase II detoxification enzymes are needed to induce their transcription. Keap-1, Kelch-like ECH-associated protein1; (ECH, erythroid cell-derived protein with CNC homology, CNC, cup ´n collar inhibitor protein); Nrf2, nuclear factor erythroid 2-related factor 2; sMaf (small protein) musculoaponeurotic fibrosarcoma oncogene, active co-transcription factor of Nrf2.

An “oxidative burst” occurs when persistent triggers such as aseptic inflammation, hypoxia, acidosis, and free radicals from pro-inflammatory macrophages and neutrophils lead to the substantial consumption of oxygen anions. The process can continue to produce oxidatively altered self-lipids. Cell detritus, the primary source of these self-antigens (DAMPs), prompts this reaction. Free radicals activate the transcription factor Nrf2, which is recruited to AREs (49). However, the release of Nrf2 from its “cup and collar” inhibitor, Kelch-like ECH-associated protein-1 (Keap-1), and the involvement of the small cellular protein c-Maf as a co-transcription factor can be reduced (**Figure 3**), thereby diminishing Nrf2´s activity (49). An overproduction of free radicals coupled with insufficient AREs can heighten the sensation of pain (50, 51). Moreover, the activation of the multi-protein inflammasome results in the production of pro-inflammatory mediators IL-1 and IL-18 and induces pyroptosis (52). Unfortunately, self-antigens can provoke inflammation reactions comparable to those triggered by xenobiotics during the initial defense step.

### 2.5 DAMPs and homeostasis

Posttraumatic inflammatory triggers multiple factors that can disrupt the physiological homeostasis of cells. This response requires a connection between the cytosol and the external environment. Among the oldest membrane channels in eukaryotes, from yeast to humans, are the transient receptor potential (TRP) cationic channels (53). These channels, which regulate responses to physical, chemical, and osmotic-sensitive stimuli, open intercellular membrane pores in both directions, allowing the passage of second messengers and small molecules. They also act as defense mechanisms when cells face threats.

TRP channels are involved in the activation of exocytosis in nerves and mast cells (MC) and in the depolarization of smooth muscle cells (SMCs), leading to relaxation and vasodilatation through the elevation of cytosolic calcium concentration [Ca^2+^]i from approximately 0.1 µM to high values (extracellular [Ca^2+^]o approximately 1 mM). This elevation triggers the exocytosis of cellular granula. Through connexins, which form gap junctions, signal transmission between SMCs can create a functional syncytium.

Additionally, the destruction of cells releases intracellular ATP ([ATP]_i_ 1–5 mM) into the interstitial space ([ATP]_o_ 10 nM), where the elevated ATP concentration serves as a DAMP signal (54) (as mentioned in subsection 2.1), previously known as the “find me molecule” (55). This activates various cells involved in inflammation and immunity. Ectonucleotidases (CD39 and CD73) initiate the rapid breakdown of ATP-releasing adenosine diphosphate (ADP), adenosine monophosphate (AMP), and adenosine, which are ligands for metabotropic G-protein-coupled receptors (P2YR, P1A1, P1A2, and P1A3) and ionotropic receptors P2XR. ATP, cAMP, PKA, free radicals, and low pH can maintain TRP-(member) V1 in an open state if ligands are present.

Simultaneously, exocytosis of neuropeptides such as ATP, calcitonin gene-related peptide (CGRP), and substance P (SP) occurs from peripheral sensory nerve endings and nerve varicosities in the microcirculation. Unlike sympathetic nerves, sensory nerves conduct impulses both antidromically and orthodromically, where antidromic conduction also leads to the release of neurotransmitters, causing vasodilation and enhancing pain sensation. In such cases, ATP and SP act as co-transmitters of CGRP. The sympathetic and sensory nerves interact via negative feedback; the activity of sensory nerves can reduce sympathetic vasoconstriction, and vice versa (56, 57). In this context, the focus is on vasodilation (hyperperfusion); therefore, further discussion of sympathetic vasoconstriction is unnecessary.

Humoral and cellular factors are intertwined in the development of trauma-triggered auto inflammation. The following subsections focus on specific cell formations with important impact for resolution and/or self-perpetuating augmentation of auto-inflammation.

### 2.6 Ambivalent behavior of mononuclear macrophages (pro-inflammatory vs. anti-inflammatory)

Different types of macrophages are present in different human tissues, including circulating blood. These macrophages directly react to the environment and support innate immunity. They exist primarily as precursor cells in tissues to maintain local homeostasis. If necessary, these cells can self-replicate using an autocrine loop with IFN-γ.

In 2001, Mills (58) observed that different ligands could polarize macrophages into different cell-physiological outputs. Soluble oxidative-modified fragments induce pro-inflammatory activity in macrophages with cytotoxic attitudes. In contrast, macrophages mutate to anti-inflammatory activity during homeostasis or aseptic wound healing. Mills adapted this classification from that of T helper cells, where T_H_1 cells are described as “host defense” and T_H_2 cells as “host repair.”

The distinction between pro-inflammatory and anti-inflammatory macrophages lies in their roles within the urea cycle (59–61). In activated pro-inflammatory macrophages, the presence of iNOS - a target gene for activated NF-κB, HIF1, and NKT cells - prompts arginine to be catalyzed by the enzyme NADPH oxidase 2 (NOX2). This consumption of free oxygen mediates the production of cytotoxic NO**˙** and citrulline (62). Conversely, in anti-inflammatory macrophages where iNOS is absent, arginine is catalyzed by arginase II into urea and ornithine, which are known for their wound healing properties (63). Consequently, in infected wounds, high levels of citrulline are observed, whereas aseptic wounds exhibit enhanced ornithine (and urea) concentrations (63). Notably, the switching from pro-inflammatory to anti-inflammatory and vice versa is autonomously managed by the macrophages without requiring the support of other cells, highlighting the pivotal roles of iNOS in regulating the urea-cycle.

**Figure 4:**
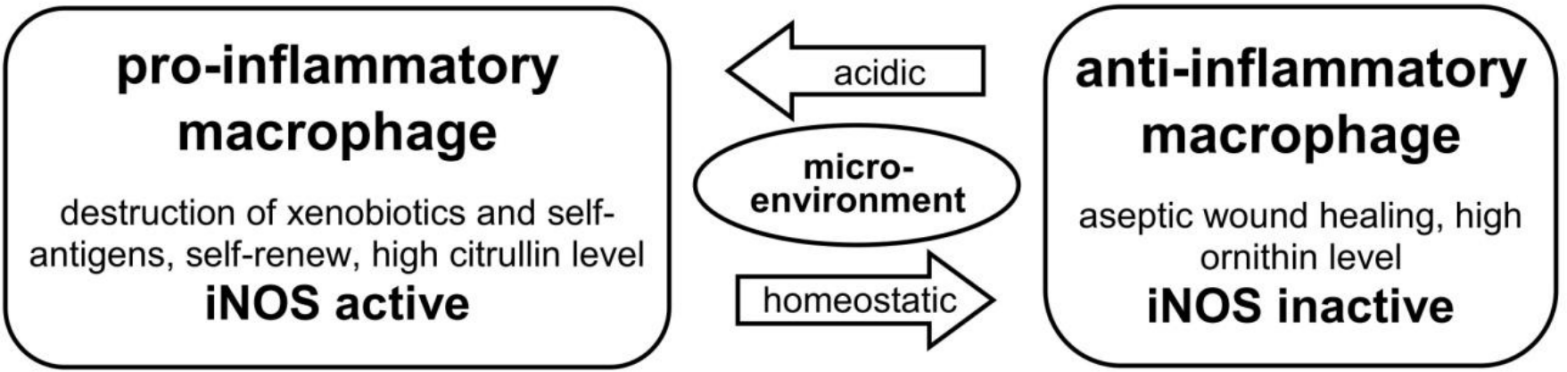
[mod. based on (20, 31, 58)]. Outline of types and functions of macrophages: Macrophages have a dichotomous functionality: dependent on their environment and the presence or absence of defined metabolic products (citrullin vs. ornithin) in the urea-cycle. They can change autonomously between cytotoxic pro-inflammatory and curative anti-inflammatory function. Pro-inflammatory macrophages show an active induced nitric oxide synthase, which is missing in anti-inflammatory macrophages during healing process.

Pro-inflammatory macrophages can also produce anti-inflammatory IL-10 in the presence of c-Maf as a co-transcription factor (64), similar to T_H_1 cells. IL-10 inhibits the polarization of T_H_0 cells into T_H_1 subset cells (65, 66) and blocks the binding of IFN-γ to its DNA target genes, due to its high affinity with IFN-γ (21, 65, 66). After the resolution of inflammation, cytotoxic pro-inflammatory macrophages can transition to curative anti-inflammatory macrophages and stabilize homeostasis. Thus, the term “ambivalent behavior” used in the headline of this subsection reflects how the physiological output of macrophages is influenced by their environment context (20, 31, 60) (**Figure 4**).

### 2.7 Mast cells

ATP, CGRP, and SP neuropeptides activate MCs, which are commonly found in the microcirculation, especially in the skin. Depending on environmental conditions such as hypoxia, these cells rapidly undergo self-development and augmentation. Their granules store pro-inflammatory mediators, including the three major pro-inflammatory cytokines (IL-1β, TNF-α, and IL-6). Additional mediators are synthesized *de novo* and released in 24–48 hours if needed (67, 68). Numerous vasoactive peptides, including histamine (H), adrenomedullin (AM), neurotensin (NT), SP, vascular endothelial growth factor (VEGF), and vasoactive intestinal peptide (VIP), support local hyperperfusion and the extravasation of immune cells and serum. Non-physiologically induced nitric oxide synthase (iNOS) is long-lived and produces substantial amounts of reactive nitrogen species (RNS), such as NO**˙**, to combat invaders. However, under certain circumstances, this species can also act against the host (see subsection 2.2).

Following the initiation of a posttraumatic auto-inflammatory reaction, the process is sustained and intensified by the transcription of cytokines, chemokines, and antimicrobials, as detailed by Punt et al. (21). The pivotal transcription factor, NF-κB, regulates over 500 target genes (see subsection 2.3) (47). Additionally, pattern recognition occurs through various cells, and innate immunity is activated. In an inflammatory environment, T-cell plasticity leads to the polarization of lymphocytes in a pro-inflammatory manner, whereas anti-inflammatory mediators are suppressed (cross-regulation) (31).

### 2.8 Local augmentation of innate immunity

In 2002, Matzinger (5) postulated in her seminal work “The Danger Model: A Renewed Sense of Self “, that the immune system is more attuned to entities causing damage rather than those that are foreign. The concept of DAMPs was first introduced in 2004 by Seong and Matzinger (6) in “Hydrophobicity: an ancient damage-associated molecular pattern that initiates innate immune responses.” They described ligands, including hyaluronan, which bind to PRRs such as TLRs on immune cell surfaces, activating innate immune reactions similar to those triggered by PAMPs with pathogenic ligands like lipopolysaccharides. Unlike PAMPs, which are exogenous, DAMPs are endogenous and originate from the body itself, often referred to as self-antigenic antigens. Since their introduction, endogenous, self-antigenic, or damage-associated (danger) ligands of PRRs have been linked to aseptic or sterile inflammatory reactions (auto-inflammation). Conversely, exogenous, non-self-antigenic, or pathogen-associated ligands on PRRs characterize PAMPs as a microbial inflammatory reaction.

Posttraumatic aseptic inflammatory reactions are recognized shortly after an incident and are linked to innate immunity. These reactions are localized around the trauma site and lack systemic consequences. It is believed that local immune cells become activated without directly connecting to the central immunological system. Therefore, it is plausible that local immunologically active cells are polarized and reside in damaged tissue and thus are not detected on routine blood examinations. Since they are not present under homeostatic conditions, it can be inferred that they are either in the final phase of development or maturation or remain inactive.

To serve as the “first line of defense” against imminent threats, these cells must be immediately activated to protect the host and the local environment as effectively as possible. T-cell plasticity (21) allows immune cells to adapt to different environments across various body tissues. While the secondary implementation of adaptive immunity is possible, it is not immediately relevant in the primary state of innate immunity.

For activated immune cells, prompt support is essential by fostering the development of other defense cells, chemotaxis, and local immune reactions (see subsection 2.9). Another vital component of the primary defense are macrophages, which reside in a premature state within the tissue. Upon activation, they aggressively respond to both self-DAMPs and PAMPs (see subsection 2.6), initiating a cascade involving other defense cells.

### 2.9 DAMPs and dNKT cell activation

The term “damage” in the acronym “DAMP” encompasses more aspects than the term “trauma” in “posttraumatic dystrophy.” Trauma involves different types of energy sources that cause damage to cells and tissues. However, apoptosis, necrosis, changes in cytosolic homeostasis, or the presence of dangerous metabolic compounds can also occur without trauma and initiate inflammatory reactions similar to trauma. Therefore, in this study, trauma and damage are used interchangeably for simplicity.

The cell wall comprises an amphiphilic double layer of fatty acids (61, 62). Traumatic lesions liberate structures and molecules that are not normally present on the wall. These function as biological signals that activate numerous defense mechanisms, including soluble receptors, such as scavenger receptors and their adapter proteins, immunoglobulins, complement compounds, opsonins, and macrophages.

The features that characterize innate immune T cells (subsection 2.8) are fulfilled by a special subset known as natural killer T (NKT) cells, which do not complete the maturation process. Several subsets with unique attributes exist, including iNKT (NKT I), dNKT (NKT II), NKT1, NKT2, NKT17, NKT_FH_, and NKT10 (69). They develop in the thymus of the fetus before birth. A subset that specifically recognizes oxPLs is the diverse repertoire of T-cell receptor (TCR) natural killer T cells, also termed dNKT cells or NKTII cells (70).

**Figure 5:**
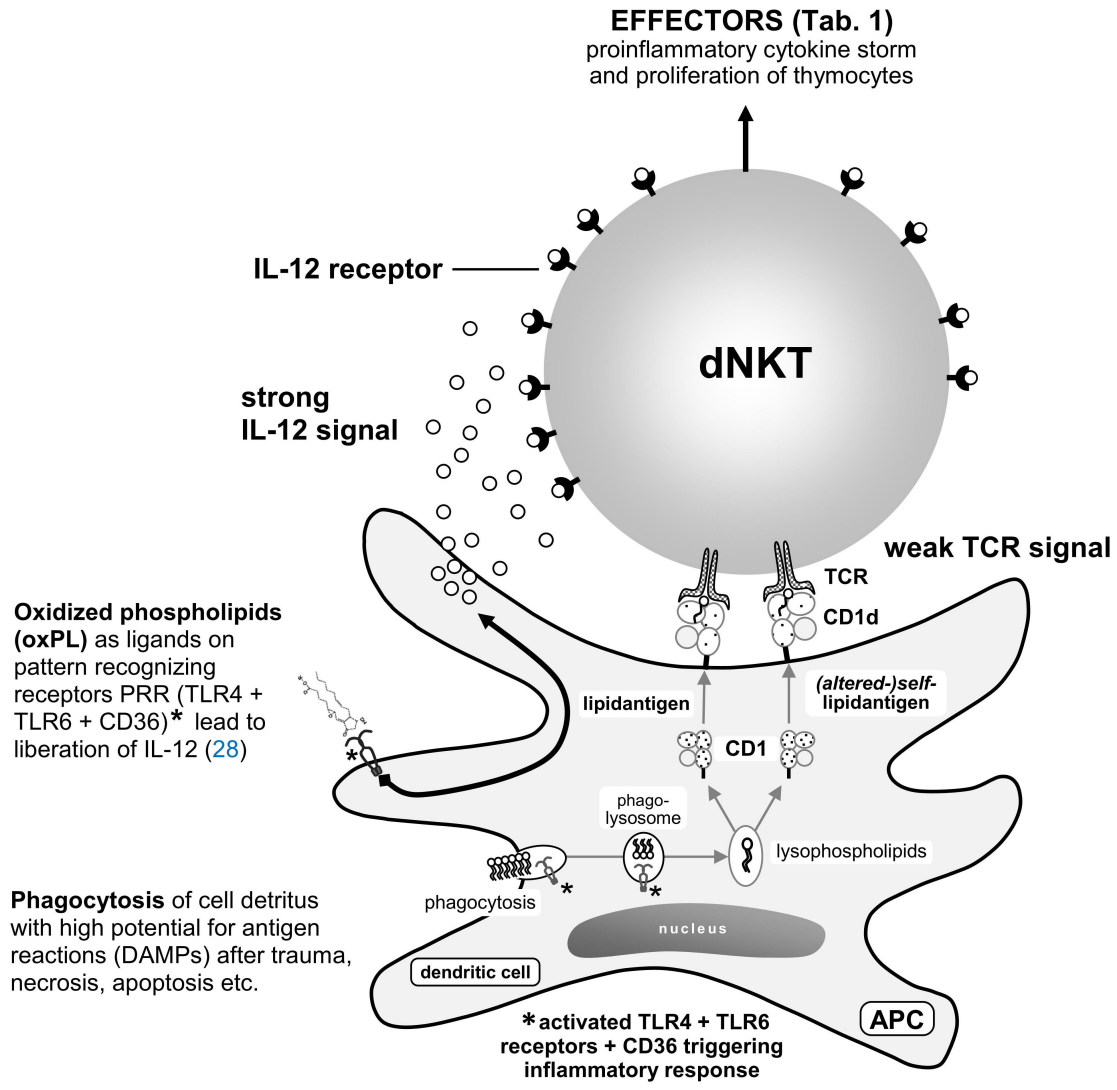
[mod. based on (74)]. During phagocytosis of posttraumatic detritus and enzymatic fragmentation in phagolysosomes with low pH values (21), phospholipases extract lipids. Polyunsaturated fatty acids PUFAs are attacked by peroxidases on the double bonds of their double acyl chains and are thus modified (**Figure 1**) (see subsection 2.2). These compounds are coupled with non-classical MHC I molecule CD1d transporter proteins [crystallography (75)]. On the surface of phagocytes (APCs) such as dendritic cells (DC), lipid compounds are present in the homing or with the blood stream passing dNKT lymphocytes [divers (reservoir of) γδ-T-cell receptor of natural killer T-cells] or iNKT lymphocytes [(semi-)invariant αβ-T-cell receptor of natural killer T-cell]. With sufficient avidity by two signaling pathways (IL-12R and TCR), activation of NKT cells occurs within 2–3 h along with fast depletion of cytokines [“cytokine storm” (76, 77)] and proliferation of other T-cells. This activity can persist during several days for epigenetic reasons (76).

Primarily composed of the γδ-version of the TCR (predominantly during prenatal development and reduced after birth), αβ-TCR iNKT cells are created prenatally and increase in adulthood (21). As part of innate immunity, they reside in border tissues, such as the skin and intestinal mucosa (71). They continuously proliferate and serve as the first line of defense against PAMPs, stimulating neutrophils, macrophages, dendritic cells, B-cells, NK cells, MHC functional cells, and self-proliferation by autocrine loop with IFN-γ. They also recognize self-molecules/DAMPs/auto-antigens, such as oxPLs, which are opsonized by antigen-presenting cells (APCs). PAMPs and DAMPs are coupled with the transporter-protein CD1d and exposed on the surface of APCs, where they undergo histocompatibility with the TCR of NKT cells (72, 73) (**Figure 5**).

TLRs are absent in NKT cells; nevertheless, their activation is induced by TCRs, IL-Rs, or both (74–76). Complete activation can occur through IL-12Rs and/or IL-18Rs from various sources or through the IL-Rs along with TCRs of NKT cells. The signals can be amplified by transcription factor activator protein-1 (AP-1) (78). Complete exocytosis of NKT cells leads to the “cytokine storm” (76, 77) (**Table 2**). Partial activation results in partial exocytosis of granules by a minor cluster of activated receptors (avidity) or by master transcription factors of T helper cells (78).

**Table 2:** [mod. based on (76)].

		EFFECTORS of activated dNKT cells
Pro-inflammatory (IL-10, IL-17 ambivalent)	cytokines	IL-2, IL-4, IL-5, IL-6, (IL-10), IL-13, (IL-17), IL-21, IFN-γ, TNFα, TNFβ (lymphotoxin), GM-CSF, TGF-β
	chemokines	CCL3 (MIP-1α), CCL4 (MIP-1β), CCL5 (RANTES), CCL11 (Eotaxin), CXCL2, CXCL10, CX3CL1 (fractalkine)
	enzyme	iNOS
cytotoxicity		Fas-Ligand, Perforin, Granzyme B
enhanced immune responses		neutrophils, B-cells, NK cells
enhanced adaptive immunity		MHC I, MHC II

Effectors of activated dNKT cells (**Figure 5**). For details please refer to the text.

*Non-standard abbreviations:* GM-CSF granulocyte-macrophage colony-stimulating factor, MIP macrophage inflammatory protein, FasL FS-7-associated surface antigen ligand.

**Figure 5** shows a schematic representation of IL-12R and TCR activation of dNKT cells through exocytosis of IL-12 and oxPL auto-antigen-presenting dendritic cells via CD1d (non-classical MHC I) (21). Complete activation occurs rapidly after the ligand/TCR signal, and the activity of dNKT cells lasts several days d (76). Only IFN-γ and IL-4 are expressed earlier, after 1 to 2 h (83). This can be explained by the acquisition of microRNA (miRNA) during development (83, 84). Therefore, the common lymphoid progenitor cell (“naïve T_H_0” cell) rapidly polarizes to produce T_H_1- and/or T_H_2-type cytokines, eliciting an early response. Lineage-specific development results in differentiation of NKT cells with distinct cytokine transcription profiles. Subsequently, plasticity was detected in T-cell lineages (20, 69, 80, 82).

Generally, it is accepted, that these special T-cells serve as a functional bridge between the instant reactivity of innate immunity (non-specific) and the delayed reactivity of adaptive immunity (specific). For specific therapy, this primarily dichotomous configuration of the inflammatory reaction must be considered.

### 2.10 T_H_1-T_H_2 cross-regulation

**Figure 6:**
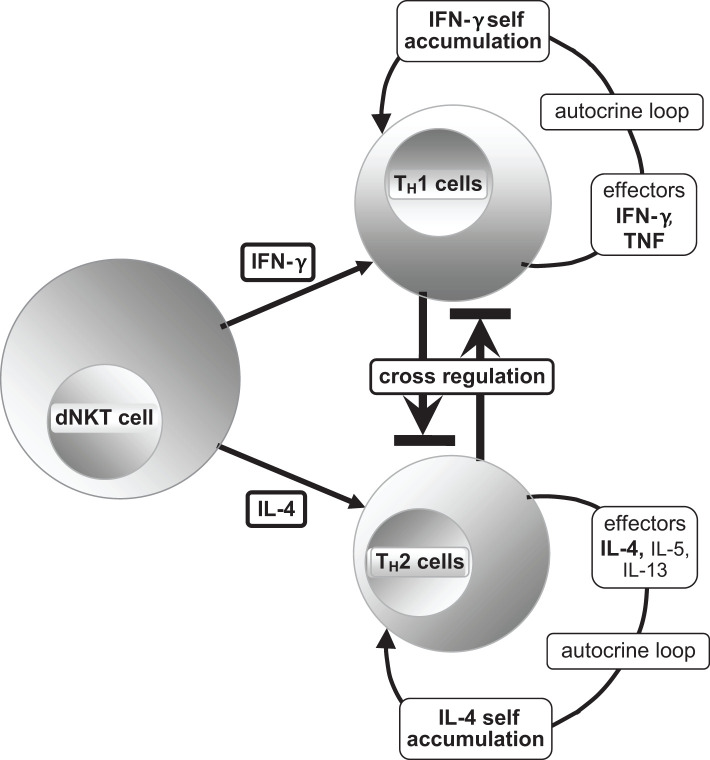
[mod. based on (21, 65, 76, 81)]. Schematic presentation of T helper subset differentiation of the common lymphoid progenitor T_H_0 to T_H_1 and T_H_2 by the effector cytokines IFN-γ and IL-4, respectively, and the production of their typical effectors with activated master transcription regulators T-bet (T_H_1) and GATA3 (T_H_2) (21, 65), reciprocal cross-regulation is possible. The T_H_1 master transcription regulator T-bet and the large protein c-Maf must work together to form a transcription factor by heterodimerization, so that induction of IL-10 is realizable without hindering the function of T-bet. Therefore, it appears to be an important condition for the resolution of inflammation and restoration of homeostasis. The same process involves the transcription factor Nrf2 for recruiting the antioxidant response elements (ARE) (23, 49, 64, 65). For alternative ways of blocking T_H_1 cells, please refer to the text.

The effector cytokine IFN-γ of dNKT cells polarizes the common lymphoid progenitor to produce T_H_1 cells and their master transcription regulator T-box expressed in T-cells (T-bet). This leads to the development of an autocrine loop (81) (**Figure 6**), where T_H_1 cells produce TNF and IFN-γ, resulting in increased differentiation of T_H_1 cells and activation of pro-inflammatory macrophages with augmentation of pro-inflammatory cytokines.

The polarizing effector for promoting T_H_2 cells is the dNKT cell effector IL-4, along with the master transcriptional regulator GATA3 [DNA sequence: (A/T)GATA(A/G)]. They block the development of T_H_1 cells indirectly (T-bet, IFN-γ, etc.) through the production of anti-inflammatory cytokines such as IL-4, IL-5, and IL-13 (**Figure 6**). dNKT cell effectors, transforming growth factor-β (TGF-β) and IL-2, support an anti-inflammatory response by polarizing the development of regulatory T (T_REG_) cells; their master transcription regulators are fork head-box-protein 3 (FOXP3) and the effector cytokines IL-10 and TGF-β. IL-10 blocks the polarization of T_H_0 cells into T_H_1 subset cells (65). Furthermore, IL-10 inhibits the binding of IFN-γ to its DNA target genes due to its high conformity with IFN-γ (21, 65, 66).

There is a positive homologous cross-regulation between T_H_17 cells and T_REG_. Both cell types exist in the skin as guardians of homeostasis and produce anti-inflammatory effectors corresponding to T_REG_. However, in an inflammatory environment, both produce pro-inflammatory effectors corresponding to the dominant T_H_17 cells. In this field, efforts are made to exert pharmacological influence to modulate aseptic inflammatory reactions (82).

### 2.11 Nuclear protein HMGB1 in innate immunity

In the early 1980s, researchers analyzing mammal cells through electrophoresis discovered chromatin proteins with rapid activity, which were later termed “high mobility group” (HMG) proteins. Years later, these proteins were found to play a role in nucleosome repair, DNA replication, and presence of the HMGB1 recombination of DNA signal sequences (RSS) (21). Furthermore, the presence of the HMGB1 enhances the efficiency of transcription factors by bending or straightening DNA signal sequences improving the precision and efficiency of the transcription process (85). The HMGB1 protein is composed of 215 amino acids, with three cysteines in different positions, each containing a sulfhydryl group. Two of these cysteines are positioned near each other, allowing the formation of a disulfide bound under certain conditions. As a redox sensitive protein, HMGB1 features two distinct functional domains: Box A, which exhibits anti-inflammatory activity (HMGA), and Box B, which exhibits pro-inflammatory activity (HMGB). Box A is a natural antagonist of Box B (86). Additionally, the subgroups HMGB2 and HMGB3, located in specific tissues, re-involved in the production of interferon type1 and pro-inflammatory mediators (87).

HMGB1 plays a crucial role in inflammatory processes. Upon modifiation by acetylation or free oxidative radicals, HMGB1 undergoes conformational changes, allowing it to shift from the nucleoplasm to cytoplasm and even to the extracellular space. Once outside the cell, it functions as damage-associated molecular pattern (DAMP) and activates innate immunity through pattern recognition receptors (PRRs) such as various Toll-like receptors (TLRs), the receptor for advanced glycation end-products (RAGE), and the G_i_-protein coupled receptor (CXCR4) located on the endo-lysosomes or cell membrane, respectively (88).

HMGB1 is produced and actively stored in the cytoplasm in its phosphorylated or methylated form by various cells, including monocytes, macrophages, immune cells, endothelial cells, platelets, and neurons within lysosomes or autophagosomes. Activated platelets, that lack a nucleus, release extracellular platelet-derived vesicles containing HMGB1 and other molecules (89). The source of this HMGB1 is assumed to depend on extramedullary megakaryocytes, which are phenotypically affected by the inflammatory environment. Normally, HMGB1 extrusion requires the fusion of cytosolic granules and the cell membrane. However, in this case of platelet-derived extracellular vesicles, interaction occurs with neutrophils, T cells, and monocytes via the P-selectin–P-selectin glycoprotein ligand1 PSGL1 pathway (89). HMGB1 can also be passively released as a result of cellular damage or during processes such as necrosis, NETosis, pyroptosis, ferroptosis, and autophagy-dependent cell death.

As mentioned before, minimal modification to the molecular structure of HMGB1 is necessary for relocalization from the nucleus to the cytoplasm and extracellular space, where it subsequently becomes “dangerous” to the innate immune system. This scenario represents HMGB1 as a prototype of a DAMP, similar to ATP, which also acts as a signal molecule (see subsection 2.5).

Excessive or uncontrolled release of HMGB1, along with activation of its receptors (such as the receptor for advanced glycated end-products [RAGE]) can lead to an increased inflammatory response. This includes oxidative bursts and cell death, characterized by increased cytokine production, inflammasome activation, pyroptosis, NETs formation, and migration of leukocytes and fibroblasts. These effects manifest as aseptic inflammation in various organs, resulting in fibrosis, sclerosis, tissue degeneration, necrosis, functional impairment, pain, and numbness if primary resolution fails (88).

### 2.12 Neutrophil extracellular trap and NETosis (neutrophil cell death)

NETs and NETosis, like HMGB1 (as mentioned in subsection 2.11), form a part of the innate immune system. Initially believed to have bactericidal effects against microbial invaders, NETs form a physical barrier through the cytosolic and extracellular de-condensation of chromatin to prevent the spread of bacteria (90). It is now understood that neutrophil elastase (NE) plays a key role in disintegrating intracellular, nuclear, and granular membranes, de-condensing chromatin, and degrading histones, which are the most abundant NET components and are potent antimicrobials. Additionally, NE is involved in the de-polymerization of actin. Furthermore, it is widely accepted that there are two types of NETs: Suicidal NETosis and Vital NETs, both occurring after the activation of neutrophil cells (91).

In Suicidal NETosis phosphorylation of the membrane-homing NADPH-complex occurs, leading to the production of free ROS. This triggers the release of peptidyl arginine deiminase 4 (PDA4) from granules, stimulating NE to move from primary (azurophilic) granules directly to the nucleus, followed by neutrophil myeloperoxidase (MPO), which like NE, is also stored in primary (azurophilic) granules. Together, nMPO and NE enhance the de-condensation of chromatin in the cytosol. Histones citrullinated by PDA4 exhibit weaker binding to DNA, further intensifying chromatin de-condensation. PDA4 and NE also generate neo-antigens that can be recognized by B and T cells, potentially inducing an autoimmune response (92). Subsequently, the neutrophil nucleus swells until the nucleoplasmic membrane ruptures, releasing de-condensed chromatin and histones, which mix with granular proteins and cytoplasm. Further swelling causes the cytoplasmic membrane to break, liberating the cytosolic contents in the form of NETs, leading to cell death, a process known as NETosis (90). De-polymerized filamentous (F-) actin is also released. As an alarmin (DAMP), F-actin contributes to the aseptic inflammatory response, similar to HMGB1, which is released from the nucleus and specific granules. Additionally F-actin serves as a specific ligand for the conventional cDC receptor DNGR-1 (dendritic cell natural killer lectin group receptor-1), with its C-type lectin-like domain (CTLD) (93), which activates multiple signaling pathways and transcription factors.

Vital NETs are induced by stimulation and activation of PAD4, which initiates the transport of NE and MPO to the nucleus, leading to the de-condensation of chromatin and histones. These components are then packaged into vesicles and transported to the extracellular space, forming NETs without causing neutrophil cell destruction, allowing the cell to maintain original functions. The extruded NETs activate additional neutrophils, perpetuating the defense process, where histones also exert toxic effects against microbial invaders. In this environment, the production of ROS and reactive nitrogen species (RNS) increases autophagy and enhances the defense activity of the NETs. Simultaneously, cell death such as NETosis releases further DAMPs (alarmins), which exacerbates the inflammatory response.

In contrast, the NADPH oxidase complex in the membrane of the cytoplasm and specific granules of activated neutrophils produces high levels of ROS in conjunction with activated inflammatory cells such as pro-inflammatory macrophages. These cells can generate significant amounts of peroxynitrite through the induction of nitric oxide synthase iNOS (subsection 2.2).

Bacterial or aseptic tissue damage is regulated by feedback mechanisms. cDC1s express the DC natural killer lectin group receptor-1 (DNGR-1), which detects tissue damage via F-actin and limits further host tissue damage by reducing neutrophil recruitment. Additionally, NETs promote the polarization of macrophages towards a reparative anti-inflammatory phenotype (93–95).

However, self-antigenic DAMPs are presented by antigen presenting cells (APCs) via the CD1d pathway (subsection 2.9) to B cells, which subsequently initiate self-antibody production. Furthermore, B cells activate platelets through CD40 and CD40L signaling. Through the P-selectin pathway, platelets and platelet-derived extracellular vesicles reduce the expression of the master transcription factor (FOXP3) in regulatory T (T_REG_) cells, thereby impairing their anti-inflammatory effects (IL-10, TGF-β). Platelets also activate NETs and NETosis connecting pro-thrombotic processes in both arterial and venous systems to responses in the microcirculation. In tissue injury, the inflammatory attributes of platelets can lead to abnormal tissue healing, such as fibrosis (89).

### 2.13 Spreading relaxation signal (clinical aspect)

Spreading relaxation signals in VSMCs are transmitted through various pathways, including connexins in a passive bidirectional manner, pannexins (40, 41), and MEGJs (38). These pathways are activated by protein kinases through processes such as phosphorylation, and nitrosylation. Channels with a diameter of 9 nm allow the passage of second messengers of ≤ 1 kDa, depending on their polarity, structure, and concentration gradient. Several arachidonates, functioning as vasodilators similar to (NO**˙**) in the microcirculation, include prostaglandin-D (PGD; MW [molecular weight]: 352 Da) (96), prostacyclin (PGI_2_; MW: 352 Da) (96), and epoxy-eicosatrienoic-acid (EET; MW: 320 Da) (96), which serves as a non-nitric oxide, non-prostanoid endothelial-derived hyperpolarization factor (EDHF) (96). These substances specifically act in arterioles and pre-capillary arteriovenous (AV) shunts with diameters ranging from 6.6–13.0 µm (97) interacting with calcium-activated potassium channels and peroxide-dependent inactivation of the heme molecule regulator of cGMP (see subsection 2.13). In mammals, CGRP is the dominant and longer-lasting vasodilator in the large arteries (57).

### 2.14 Conducted vasodilation (clinical aspect)

The spread of the aseptic inflammation following peripheral trauma is noteworthy, as the aseptic inflammatory reaction extends more peripherally than centrally. Signals from peripheral tissues, resembling those from capillary hypoxia, pass through ECs when feed arteries require a signal for vasodilatation (CVD). Signal transduction is facilitated by activated bidirectional intercellular channels (connexins), which allow the exchange of messenger molecules with a mass less than 1 kDa (98, 99). Arterial blood flow supports the peripheral expansion of the inflammation to the acral region, even if this region is not directly involved in the trauma. The central expansion of inflammation, though less clearly demarcated, appears to coincide with the transition to a homeostatic environment.

Interestingly, the diffusion of Ca^2+^ signal molecules can be visualized along the endothelium using a fluorescent Ca^2+^ sensitive dye (100), allowing for an *in vivo* representation of signal transport and a deep understanding of the immunological processes involved in aseptic inflammation. For instance, bone involvement becomes more apparent when considering the robust blood supply to the microcirculation of joint-forming bones and the resulting consequences, such as demineralization. Hypoxia, induced by open AV shunts leads to an increase in free radicals and acidosis (low pH), which enhances osteoclast (101, 102) and inhibits osteoblast activity. Osteoclasts, often referred to as the macrophages of bones (103), exhibit increased activity as pH decreases, a characteristic observed in all myeloid cell types (104–106).

### 2.15 Vascular smooth muscle cells and AV-shunts (clinical aspect)

VSMCs in the peripheral microcirculation belong to the “*single-unit type*” and are activated through cell-to-cell signal transport (connexins). However, the process of contraction and relaxation in VSMCs is much slower than that in striated muscles (100–1.000 times slower) (107). This is due to the different structure and reduced activity of myosin-ATPase and myosin-phosphatase. Furthermore, contraction begins only when the activity of myosin light chain kinase increases, which is another slow process. Differences also arise in the changes in functional influences as VSMCs adapt to physiological demands. Finally, only minor concentrations of vasodilators are required to maintain a continuous state of relaxation in the microcirculation with open AV shunts. Therefore, VSMCs can lose physiologically important basis tension (dynamic equilibrium of myosin light chain kinase [MLCK] and myosin light chain phosphatase [MLCP]) (107) and acquire ground tension, allowing them to escape sympathetic vasoconstriction (108) without losing their original function. To resolve complete relaxation (paralysis) under physiological conditions MLCP must be inhibited.

MLCP is blocked by the protein kinase C (PKC) pathway. When the G-protein-coupled receptor α_q_ is activated, phospholipase Cβ (PLCβ) releases inositol-triphosphate from membrane lipids, mobilizing Ca^2+^ from the endoplasmic reticulum, leading to the activation of diacylglycerin and membrane-bound PKC. This phosphorylated protein kinase then potentiates the protein-phosphatase-1 inhibitor of 17 kDa (CPI-17), which blocks MLCP (108–110). Another blockade of MLCP is induced by the G-protein-coupled receptor α_12/13_ and activation of the RhoA-kinase ROCK, which also phosphorylates CPI-17 (111).

Complete blockade of MLCK and MLCP causes reversible paralysis of SMCs, which persists with permanent activation of G-protein-coupled receptors of vasodilators, including AM, CGRP, adenosine, PGI_2_, PGE_2_, PGD, VIP, and histamine H_1_, even at low concentrations. These vasodilators are present in aseptic inflammation reactions and in transcription or biological processes liberated from granules of sensory nerves, or MCs. Excess ATP and cAMP/PKA are transferred to adjacent VSMCs via connexins. Furthermore, diffusible NO**˙** relaxes VSMCs, facilitating the transmission of produced cGMP/PKG through connexins to neighboring VSMCs to support relaxation (39, 112). Furthermore, in the presence of superoxides and peroxides, the heme molecule within the sGC enzyme is inactivated, leading to a blockade of the negative feedback to NO**˙** and cGMP. This results in prolonged production of cGMP and PKG, further enhancing the relaxation of VSMCs (see subsections 2.2 and 2.11) (44).

Due to their different structures and actin/myosin compositions, VSMCs have a greater distention and contracture rate than striated muscles (approximately 30%) (107, 110, 113). Paralytic dilation of VSMCs dramatically enhances perfusion with hypoxic capillaries in the microcirculation and open AV shunts. This could explain the “elasticity” of the local swelling like a tight sponge of rubber provoked by hyperperfusion (further discussion in subsection 2.14).

Fine regulation of microcirculation (feed arteries, venules, and lymphatic tubules) can be modulated in different ways. In posttraumatic or aseptic inflammatory reactions, influences dominate NO**˙**, PGI_2_, nucleotides, adenosine compounds, and partially neuropeptides, like CGRP and SP.

Ions alter the polarity of membranes by exchanging Ca^2+^ and K^+^ through ion-channels and influence VSMC relaxation (35–38, 111, 112, 114) (**Figure 2**). Of importance are the AV shunts possessing widths similar to arterioles with (nearly) circular VSMCs and the pre-capillary sphincters, which are formed by helix-like bent VSMCs at the beginning of the capillaries (VSMC dimensions: approximately 100 µm in length; 6.4 ± 2.1 µm in width) (97, 107, 108). Induced dilatation of VSMCs in the microcirculation reduces capillary perfusion due to increased resistance to blood flow, micro-thrombosis by TNF, and elevated interstitial pressure (22). Owing to AV shunts, bypasses result in oxygenated blood flow passing directly into the venous vascular system. Venous oxygen pressure increases depending on the number of AV shunts that persist. Local acidosis by hypoxia mobilizes redox-sensitive transcription factors (see subsection 2.3) and activates vasodilators (see subsection 2.11) and innate immune cells (see subsections 2.8 and 2.9). Furthermore, apoptosis occurs, creating free radicals and DAMP.

### 2.16 Skin (clinical aspect)

Besides hyperpathia and allodynia, chronic-aseptic inflammatory reactions cause hyperhidrosis and hypertrichosis. Due to the same connexins (Cx 40) found in VSMCs and ECs (38, 40, 41), the vascular proliferation of aseptic auto-inflammation can be observed in the skin through the traditional symptoms of Celsus and Galen. It can be assumed that stimulation of secretory glands and hair follicles occurs because several growth factors are synthesized *de novo* by activated MCs (67, 68). Simultaneously, the proliferation and maturation of MC are induced. Stimulated sensory nerve endings produce nerve growth factor (NGF), which supports an inflammatory reaction similar to that of nucleotides and adenosine compounds.

Correctly interpreting the classical symptoms of inflammation in PTD is invaluable. First, the symptom “redness” (*rubor*) does not mean “hyperemia” in the traditional sense, but widened AV shunts with paralyzed VSMCs and elevated perfusion rates; oxygenated blood returns directly to the venous system, causing the skin color to turn red due to “hyper-oxygenized” venous microcirculation. Second, “swelling” (*tumor*) does not refer to edema, such as cardiac venous insufficiency, but to hyperperfusion with widened AV shunts; the consistency is elastic, similar to that of a tight rubber sponge. Third, “heat” (*calor*) refers to hyperperfusion of core-tempered arterial blood.

Capillary hypoxia leads to the production of free radicals, acidosis, and oxPLs. These DAMPs activate TRP-A1 ion-channels in sensory nerves, intensifying the sensation and duration of pain (50, 51). Therefore, the fourth symptom described by Celsus “pain” (*dolor*), is a consequence of aseptic auto-inflammation and not its cause (7).

## 3 Discussion

The etiology of PTD in extremities has historically been referred to as “slipped healing-inflammation,” a term first defined by Sudeck in 1901 (1). This concept stemmed from clinical observations that each trauma exhibited signs of aseptic inflammation prior to healing, as described by Celsus and Galen nearly 2,000 years earlier. In cases of PTD, the transformation from inflammation to regeneration is incomplete, often resulting in the perpetuation of the condition and ultimately leading to fibrosis of soft tissues and functional loss in joints and extremities. The specific steps of this transformation remain a subject of ongoing discussion. Over the past two decades, remarkable advances in immunology have shed light on these processes, offering potential resolutions to clinical challenges such as posttraumatic dystrophy.

The initial consequences of trauma include damage to cell walls, releasing substantial amounts of unusual moieties known as damage-associated molecular patterns (DAMPs), collectively referred to as debris that must be cleared. This triggers the activation of innate immunity to restore the micro-environment, exhibiting the traditional signs of inflammation.

Under physiological conditions, healing typically occurs within 10–14 days. However, local ischemia and hypoxia can prolong the healing process, leading to the development of acidosis and an augmented local innate immunity response. As outlined in section 2, tissue damage exacerbates through cell necrosis and apoptosis creating an acidic milieu akin to microbial infection. Activated histocompatibility primarily occurs between APC (such as dendritic cells, neutrophils, and B-cells) and local resident natural killer T cells (NKT cells). These function like a turbo-machinery in innate immunity-reactions with “cytokine storm,” cytotoxicity and recruitment of lymphocytes. Given that host debris triggers innate immunity-reactions, the term “auto-inflammation” is aptly used. The outcome - whether healing or Posttraumatic Dystrophy - typically becomes apparent within 2–4 weeks and can extend up to 6 to 8 weeks after trauma.

As long as the sterile inflammation process continues, free radicals are produced, perpetuating local auto-inflammation reactions. The spread of auto-inflammation extends along the endothelium of vessels until a physiological environment is reached, as detailed in subsection 2.11 f. The creation of AV shunts allows hypoxia to persist in the capillary bed, where the endothelium continues to produce more free radicals.

Sensory nerves are also implicated in this auto-inflammation process. Notably, patients had no pain in the affected region before the injury. Persistent pain after a traumatic incident primarily results from the activation of TRPA1 and other TRP channels in the presence of sterile auto-inflammation. This activation induces neuronal nitric oxide synthase, which produces nitric oxide and, in conjunction with superoxide, forms the RNS peroxynitrite. Both hypoxia and acidic environments further activate TRP channels.

Recovery is marked by an enhanced venous pO_2_ and amelioration of microcirculation disturbances in patients. The challenge of therapy is multi-faceted, and treating a single symptom does not always lead to therapeutic success. Prophylaxis seems to be a more effective approach to prevent this complication, as causal therapy remains elusive. In fact, with the increasing commonality of osteosynthesis for fractures, functional after-treatment of the extremity that supports microcirculation leads to a better outcome and few instances of posttraumatic dystrophy. The beginning of prophylaxis is a key finding from decades of clinical research.

In 2022 Matzinger (30) published a notable work providing an immunological perspective on this topic. The complete signaling cascade involving panoply of mediators and immune cells with defined defense activities is referred to as “effector class” (tissue-based class control). This DAMP-mediated auto-inflammation, triggered by trauma and exacerbated by auto-reactive T cells, is characterized as “immensely destructive to the tissue”. Auto-antibodies against DAMPs, induced by activated B-1 B-cells, sustain immune responses longer than necessary, leading to a persistent and self-perpetuating cycle. This understanding opens new avenues for exploring the etiology of posttraumatic dystrophy.

## 4 Summary

There are remarkable similarities between the activities of auto-reactive T cells in innate immunity and PTD. Therefore, it is more convincing than contradictory to deduce a relationship: the etiology of PTD as an aseptic auto-inflammatory reaction becomes clearer in the broad field of posttraumatic innate immunity with local spreading properties in all affected tissues.

## 5 Further directions

Determination of proinflammatory mediators, auto-antibodies, auto-antigens, auto-antioxidants, isoprostanes as DAMP and lymphocyte specific probes are the first evaluations with the option for further detailed screening in PTD patients.

## Data Availability

The original contributions presented in the study are included in the article/supplementary material. Further inquiries can be directed to the corresponding author.

## References

[1] Sudeck P. Über die akute (reflektorische) Knochenatrophie nach Entzündungen und Verletzungen an den Extremitäten und ihre klinischen Erscheinungen, in: *Fortschr a d Geb d Röntgenstrahlen* (1901–1902) . Hamburg: Verlag Lucas Gräfe & Sillem. Available online at: https://archive.org/details/fortschritte-rontgenstrahlen-5-6 (Accessed September 17, 2024).

[2] Talmage DW. A century of progress: beyond molecular immunology. J Immunol. (1988) 141:S5–S16. doi: 10.4049/jimmunol.141.7.5 3049812

[3] Janeway CA. Approaching the asymptote? Evolution and revolution in immunology. Cold Spring Harb Symp Quant Biol. (1989) 54:1–13. doi: 10.1101/sqb.1989.054.01.003 2700931

[4] Matzinger P. Tolerance, danger, and the extended family. Annu Rev Immunol. (1994) 12:991–1045. doi: 10.1146/annurev.iy.12.040194.005015 8011301

[5] Matzinger P. The danger model: A renewed sense of self. Science. (2002) 296:301–5. doi: 10.1126/science.1071059 11951032

[6] Seong S-Y Matzinger P. Hydrophobicity: an ancient damage-associated molecular pattern that initiates innate immune responses. Nat Rev Immunol. (2004) 4:469–78. doi: 10.1038/nri1372 15173835

[7] Sudeck P. Die sogenannte akute Knochendystrophie als Entzündungsvorgang. Chirurg. (1942) 14:449–58.

[8] Evans JA. Reflex sympathetic dystrophy. Surg Clin North Am. (1946) 26:780–90. 20988032

[9] Oehlecker F. Zu der Bezeichnung “Sudecksches Syndrom” oder kurz “Sudeck”. [About the term Sudeck´s syndrome or Sudeck for short. Chirurg. (1948) 19:398–403. 18893348

[10] Blumensaat C. Der heutige Stand der Lehre vom Sudeck-Syndrom [The present extent of knowledge of the Sudeck syndrome]. In: Hefte Unfallheilkd, vol. 51. Springer, Berlin, Göttingen, Heidelberg (1956). p. 1–225. 13331264

[11] Merskey H Bogduk N eds. Classification of chronic pain: descriptions of chronic pain syndromes and definitions of pain terms. 2nd ed. Seattle: IASP Press (1994) p. 40–2.

[12] Coderre TJ. Complex regional pain syndrome: what’s in a name? J Pain. (2011) 12:2–12. doi: 10.1016/j.jpain.2010.06.001 20634146 PMC4850066

[13] Harden RN Oaklander AL Burton AW. Complex regional pain syndrome: practical diagnostic and treatment guidelines, 4th ed. Pain Med. (2013) 14:180–229. doi: 10.1111/pme.12033 23331950

[14] Birklein F Dimova V. Complex regional pain syndrome-up-to-date. Pain Rep. (2017) 2(6):e624. doi: 10.1097/PR9.0000000000000624 29392238 PMC5741324

[15] Okumo T Takayama Y Maruyama K Kato M Sunagawa M. Senso-immunologic prospects for complex regional pain syndrome treatment. Front Immunol. (2022) 12:786511. doi: 10.3389/fimmu.2021.786511 35069559 PMC8767061

[16] Stanton-Hicks M Jänig W Hassenbusch S Haddox JD Boas R Wilson P. Reflex sympathetic dystrophy: changing concepts and taxonomy. Dt Aerztebl. (1995) 63:127–33. 10.1016/0304-3959(95)00110-E 8577483

[17] Scola A Scola E. Posttraumatic dystrophy. diagnosis and therapy after distal radius fractures and hand injuries. Unfall. (2013) 116:723–32. doi: 10.1007/s00113-013-2450-x 23918032

[18] Scola A Scola E. Bone resorption in posttraumatic dystrophy. Root cause analysis based on the literature. Unfall. (2014) 117:957–61. doi: 10.1007/s00113-014-2643-y 25274392

[19] Scola A Scola E. Reliability of venous blood gas analysis and radionuclide angiography in post-traumatic dystrophy. Unfall. (2017) 120:501–8. doi: 10.1007/s00113-017-0341-2 28275848

[20] Mills CD Kincaid K Alt JM Heilman MJ Hill AM. M-1/M-2 macrophages and the Th1/Th2 paradigm. J Immunol. (2000) 164:6166–73. doi: 10.4049/jimmunol.164.12.6166 10843666

[21] Punt J Stranford SA Jones PP Owen JA eds. *Kuby* Immunology Macmillan education. 8th ed. New York: W. H. Freeman and Company (2019).

[22] Eltzschig HK Carmeliet P. Hypoxia and inflammation. N Engl J Med. (2011) 364:656–65. doi: 10.1056/NEJMra0910283 PMC3930928 21323543

[23] Kasai S Shimizu S Tatara Y Mimura J Itoh K. Regulation of Nrf2 by mitochondrial reactive oxygen species in physiology and pathology. Biomolecules. (2020) 10:320. doi: 10.3390/biom10020320 32079324 PMC7072240

[24] Freigang S. The regulation of inflammation by oxidized phospholipids. Eur J Immunol. (2016) 46:1818–25. doi: 10.1002/eji.201545676 27312261

[25] Tyurina YY St. Croix CM Watkins SC Watson AM Epperly MW Anthonymuthu TS. Redox (phospho)lipidomics of signaling in inflammation and programmed cell death. J Leukoc Biol. (2019) 106:57–81. doi: 10.1002/JLB.3MIR0119-004RR 31071242 PMC6626990

[26] Binder CJ Papac-Milicevic N Witztum JL. Innate sensing of oxidation-specific epitopes in health and disease. Nat Rev Immunol. (2016) 16:485–97. doi: 10.1038/nri.2016.63 PMC7097710 27346802

[27] Schieber M Chandel NS. ROS function in redox signaling and oxidative stress. Curr Biol. (2014) 24:R453–62. doi: 10.1016/j.cub.2014.03.034 PMC4055301 24845678

[28] Rocha DM Caldas AP Oliveira LL Bressan J Hermsdorff HH. Saturated fatty acids trigger TLR4-mediated inflammatory response. Atherosclerosis. (2016) 244:211–5. doi: 10.1016/j.atherosclerosis.2015.11.015 26687466

[29] Milne GL Yin H Hardy KD Davies SS Roberts LJ. Isoprostane generation and function. Chem Rev. (2011) 111:5973–96. doi: 10.1021/cr200160h PMC3192249 21848345

[30] Matzinger P. Autoimmunity: are we asking the right question? Front Immunol. (2022) 13:864633. doi: 10.3389/fimmu.2022.864633 36405714 PMC9671104

[31] Serbulea V DeWeese D Leitinger N. The effect of oxidized phospholipids on phenotypic polarization and function of macrophages. Free Radic Biol Med. (2017) 111:156–68. doi: 10.1016/j.freeradbiomed.2017.02.035 PMC5511074 28232205

[32] Krönke G Leitinger N. Oxidized phospholipids at the interface of innate and adaptive immunity. Future Lipidol. (2006) 1:623–30. doi: 10.2217/17460875.1.5.623

[33] Hazen SL. Oxidized phospholipids as endogenous pattern recognition ligands in innate immunity. J Biol Chem. (2008) 283:15527–31. doi: 10.1074/jbc.R700054200 PMC2414290 18285328

[34] Görlach A Dimova EY Petry A Martínez-Ruiz A Hernansanz-Agustín P Rolo AP. Reactive oxygen species, nutrition, hypoxia and diseases: problems solved? Redox Biol. (2015) 6:372–85. doi: 10.1016/j.redox.2015.08.016 PMC4565025 26339717

[35] Boedtkjer E Praetorius J Matchkov VV Stankevicius E Mogensen S Füchtbauer AC. Disruption of Na^+^, HCO_3_¯ cotransporter NBCn1 (slc4a7) inhibits NO-mediated vasorelaxation, smooth muscle Ca^2+^ sensitivity, and hypertension development in mice. Circulation. (2011) 124:1819–29. doi: 10.1161/CIRCULATIONAHA.110.015974 21947296

[36] Hibino H Inanobe A Furutani K Murakami S Findlay I Kurachi Y. Inwardly rectifying potassium channels: their structure, function, and physiological roles. Physiol Rev. (2010) 90:291–366. doi: 10.1152/physrev.00021.2009 20086079

[37] Park WS Han J Earm YE. Physiological role of inward rectifier K^+^ channels in vascular smooth muscle cells. Pflugers Arch. (2008) 457:137–47. doi: 10.1007/s00424-008-0512-7 18437413

[38] Ledoux J Werner ME Brayden JE Nelson MT. Calcium-activated potassium channels and the regulation of vascular tone. Physiology. (2006) 21:69–78. doi: 10.1152/physiol.00040.2005 16443824

[39] Hofmann F. The biology of cyclic GMP-dependent protein kinases. J Biol Chem. (2005) 280:1–4. doi: 10.1074/jbc.R400035200 15545263

[40] Meșe G Richard G White TW. Gap junctions: basic structure and function. J Invest Dermatol. (2007) 127:2516–24. doi: 10.1038/sj.jid.5700770 17934503

[41] Willebrords J Crespo Yanguas S Maes M Decrock E Wang N Leybaert L. Connexins and their channels in inflammation. Crit Rev Biochem Mol Biol. (2016) 51:413–39. doi: 10.1080/10409238.2016.1204980 PMC5584657 27387655

[42] Förstermann U Münzel T. Endothelial nitric oxide synthase in vascular disease: from marvel to menace. Circulation. (2006) 113:1708–14. doi: 10.1161/CIRCULATIONAHA.105.602532 16585403

[43] Kibbe M Billiar T Tzeng E. Inducible nitric oxide synthase and vascular injury. Cardiovasc Res. (1999) 43:650–7. doi: 10.1016/s0008-6363(99)00130-3 10690336

[44] Hobbs AJ Stasch JP. Soluble Guanylat cyclase: allosteric activation and redox regulation. In: Ignarro LJ, editor. Nitric oxide: biology and pathobiology, 2nd ed. Academic Press Elsevier, London (2010). p. 301–26.

[45] Faustman D Davis M. TNF receptor 2 pathway: drug target for autoimmune diseases. Nat Rev Drug Discov. (2010) 9:482–93. doi: 10.1038/nrd3030 20489699

[46] Hellweg CE Spitta LF Henschenmacher B Diegeler S Baumstark-Khan C. Transcription factors in the cellular response to charged particle exposure. Front Oncol. (2016) 6:61. doi: 10.3389/fonc.2016.00061 27047795 PMC4800317

[47] Gilmore Lab Boston University, Biology Department. Gene resources, NF-κB target genes (2023). Available online at: http://www.bu.edu/nf-kb/gene-resources/target-genes (Accessed September 17, 2024).

[48] Schödel J Oikonomopoulos S Ragoussis J Pugh CW Ratcliffe PJ Mole DR. High-resolution genome-wide mapping of HIF-binding sites by ChIP-seq. Blood. (2011) 117:e207–17. doi: 10.1182/blood-2010-10-314427 PMC3374576 21447827

[49] Tonelli C Chio IIC Tuveson A. Transcriptional regulation by Nrf2. Antioxid Redox Signal. (2018) 29:1727–45. doi: 10.1089/ars.2017.7342 PMC6208165 28899199

[50] Liu B Tai Y Caceres AI Achanta S Balakrishna S Shao X. Oxidized phospholipid OxPAPC activates TRPA1 and contributes to chronic inflammatory pain in mice. PloS One. (2016) 11:e0165200. doi: 10.1371/journal.pone.0165200 27812120 PMC5094666

[51] Oehler B Kistner K Martin C Schiller J Mayer R Mohammadi M. Inflammatory pain control by blocking oxidized phospholipid-mediated TRP channel activation. Sci Rep. (2017) 7:5447. doi: 10.1038/s41598-017-05348-3 28710476 PMC5511297

[52] Groslambert M Py BF. Spotlight on the NLRP3 inflammasome pathway. J Inflam Res. (2018) 11:359–74. doi: 10.2147/JIR.S141220 PMC6161739 30288079

[53] Voolstra O Huber A. Post-translational modifications of TRP channels. Cells. (2014) 3:258–87. doi: 10.3390/cells3020258 PMC4092855 24717323

[54] Shaw ER Matzinger P. Transient autoantibodies to danger signals. Front Immunol. (2023) 14:1046300. doi: 10.3389/fimmu.2023.1046300 36742299 PMC9889632

[55] Elliott MR Chekeni FB Trampont PC Lazarowski ER Kadl A Walk SF. Nucleotides released by apoptotic cells act as a find-me signal to promote phagocytic clearance. Nature. (2009) 461:282–6. doi: 10.1038/nature08296 PMC2851546 19741708

[56] Westcott EB Segal SS. Perivascular innervation: A multiplicity of roles in vasomotor control and myoendothelial signaling. Microcirculation. (2013) 20:217–38. doi: 10.1111/micc.12035 PMC3771679 23289720

[57] Russell FA King R Smillie SJ Kodji X Brain SD. Calcitonin gene-related peptide: physiology and pathophysiology. Physiol Rev. (2014) 94:1099–142. doi: 10.1152/physrev.00034.2013 PMC4187032 25287861

[58] Mills CD. Macrophage arginine metabolism to ornithine/urea or nitric oxide/citrulline: a life or death issue. Crit Rev Immunol. (2001) 21:399–425. doi: 10.1615/CritRevImmunol.v21.i5.10 11942557

[59] Ley K. M1 means kill; M2 means heal. J Immunol. (2017) 199:2191–3. doi: 10.4049/jimmunol.1701135 28923980

[60] Buscher K Ehinger E Gupta P Pramod AB Wolf D Tweet G. Natural variation of macrophage activation as disease-relevant phenotype predictive of inflammation and cancer survival. Nat Commun. (2017) 8:16041. doi: 10.1038/ncomms16041 28737175 PMC5527282

[61] Berg JM Tymoczko JL Stryer L eds. Biochemie. Heidelberg: Springer Spektrum (2013). Corrected reprint (2014), 7th ed. Translation from American edition: Berg JM, Tymoczko JL, Stryer L, editors. Gatto Jr. GJ, co-editor. Biochemistry, Seventh ed. (2012), New York, W. H. Freeman and Company.

[62] Müller-Esterl W ed. Biochemie. 3rd ed. Berlin: Springer Spektrum (2018).

[63] Wu G Morris SM. Arginine metabolism: nitric oxide and beyond. Biochem J. (1998) 336:1–17. doi: 10.1042/bj3360001 9806879 PMC1219836

[64] Cao S Liu J Song L Ma X. The protooncogene c-Maf is an essential transcription factor for IL-10 gene expression in macrophages. J Immunol. (2005) 174:3484–92. doi: 10.4049/jimmunol.174.6.3484 PMC2955976 15749884

[65] Mitchell RE Hassan M Burton BR Britton G Hill EV Verhagen J. IL-4 enhances IL-10 production in Th1 cells: implications for Th1 and Th2 regulation. Sci Rep. (2017) 7:11315. doi: 10.1038/s41598-017-11803-y 28900244 PMC5595963

[66] Windsor WT Syto R Tsarbopoulos A Zhang R Durkin J Baldwin S. Disulfide bond assignments and secondary structure analysis of human and murine interleukin 10. Biochemistry. (1993) 32:8807–15. doi: 10.1021/bi00085a011 8364028

[67] Sismanopoulos N Delivanis D-A Alysandratos K-D Angelidou A Therianou A Kalogeromitros D. Mast cells in allergic and inflammatory diseases. Curr Pharm Des. (2012) 18:2261–77. doi: 10.2174/138161212800165997 22390690

[68] Theoharides TC Alysandratos K-D Angelidou A Delivanis D-A Sismanopoulos N Zhang B. Mast cells and inflammation. Biochim Biophys Acta. (2012) 1822:21–33. doi: 10.1016/j.bbadis.2010.12.014 21185371 PMC3318920

[69] Kim EY Lynch L Brennan PJ Cohen NR Brenner MB. The transcriptional programs of iNKT cells. Semin Immunol. (2015) 27:26–32. doi: 10.1016/j.smim.2015.02.005 25841627 PMC6322908

[70] Tatituri RVV Watts GFM Bhowruth V Barton N Rothchild A Hsu FF. Recognition of microbial and mammalian phospholipid antigens by NKT cells with diverse TCRs. Proc Natl Acad Sci U S A. (2013) 110:1827–32. doi: 10.1073/pnas.1220601110 PMC3562825 23307809

[71] Muro R Takayanagi H Nitta T. T cell receptor signaling for γδT cell development. Inflammation Regener. (2019) 39:6. doi: 10.1186/s41232-019-0095-z PMC6437992 30976362

[72] Barral DC Brenner MB. CD1 antigen presentation: how it works. Nat Rev Immunol. (2007) 7:929–41. doi: 10.1038/nri2191 18037897

[73] Brigl M Brenner MB. How invariant natural killer T cells respond to infection by recognizing microbial or endogenous lipid antigens. Semin Immunol. (2010) 22:79–86. doi: 10.1016/j.smim.2009.10.006 19948416

[74] Brigl M Tatituri RVV Watts GFM Bhowruth V Leadbetter EA Barton N. Innate and cytokine-driven signals, rather than microbial antigens, dominate in natural killer T cell activation during microbial infection. J Exp Med. (2011) 208:1163–77. doi: 10.1084/jem.20102555 PMC3173255 21555485

[75] Rossjohn J Pellicci DG Patel O Gapin L Godfrey DI. Recognition of CD1d-restricted antigens by natural killer T cells. Nat Rev Immunol. (2012) 12:845–57. doi: 10.1038/nri3328 PMC3740582 23154222

[76] Kohlgruber AC Donado CA LaMarche NM Brenner MB Brennan PJ. Activation strategies for invariant natural killer T cells. Immunogenetics. (2016) 68:649–63. doi: 10.1007/s00251-016-0944-8 PMC5745583 27457886

[77] Clark IA Vissel B. The meteorology of cytokine storms, and the clinical usefulness of this knowledge. Semin Immunopathol. (2017) 39:505–16. doi: 10.1007/s00281-017-0628-y PMC5495849 28451786

[78] Engel I Kronenberg M. Transcriptional control of the development and function of Vα14i NKT cells. Curr Top Microbiol Immunol. (2014) 381:51–81. doi: 10.1007/82_2014_375 24839184

[79] Brennan PJ Brigl M Brenner MB. Invariant natural killer T cells: an innate activation scheme linked to diverse effector functions. Nat Rev Immunol. (2013) 13:101–17. doi: 10.1038/nri3369 23334244

[80] O’Shea JJ Paul WE. Mechanisms underlying lineage commitment and plasticity of helper CD4+ T cells. Science. (2010) 327:1098–102. doi: 10.1126/science.1178334 PMC2997673 20185720

[81] Hung JT Huang JR Yu AL. Tailored design of NKT-stimulatory glycolipids for polarization of immune responses. J BioMed Sci. (2017) 24:22. doi: 10.1186/s12929-017-0325-0 28335781 PMC5364570

[82] Awasthi A Kuchroo VK. Th17 cells: from precursors to players in inflammation and infection. Int Immunol. (2009) 21:489–98. doi: 10.1093/intimm/dxp021 PMC2675030 19261692

[83] Stetson DB Mohrs M Reinhardt RL Baron JL Wang ZE Gapin L. Constitutive cytokine miRNAs mark natural killer (NK) and NK T cells poised for rapid effector function. J Exp Med. (2003) 198:1069–76. doi: 10.1084/jem.20030630 PMC2194220 14530376

[84] Godfrey DI Kronenberg M. Going both ways: immune regulation via CD1d-dependent NKT Cells. J Clin Invest. (2004) 114:1379–88. doi: 10.1172/JCI23594 PMC525753 15545985

[85] Postnikov YV Bustin M. Functional interplay between histone H1 and HMG proteins in chromatin. Biochim Biophys Acta. (2016) 1859:462–7. doi: 10.1016/j.bbagrm.2015.10.006 PMC4852864 26455954

[86] Bianchi ME Manfredi AA. High-mobility group box 1 (HMGB1) protein at the crossroads between innate and adaptive immunity. Immunol Rev. (2007) 220:35–46. doi: 10.1111/j.1600-065X.2007.00574.x 17979838

[87] Castiglioni A Canti V Rovere-Querini P Manfredi AA. High-mobility group box 1 (HMGB1) as a master regulator of innate immunity. Cell Tissue Res. (2011) 343:189–99. doi: 10.1007/s00441-010-1033-1 20835834

[88] Yuan JY Guo L Ma JT. HMGB1 as an extracellular pro−inflammatory cytokine: Implications for drug−induced organic damage. Cell Biol Toxicol. (2024) 40:55. doi: 10.1007/s10565-024-09893-2 39008169 PMC11249443

[89] Scherlinger M Richez C Tsokos GC. The role of platelets in immune-mediated inflammatory diseases. Nat Rev Immunol. (2023) 23:495–510. doi: 10.1038/s41577-023-00834-4 36707719 PMC9882748

[90] Brinkmann V Reichard U Goosmann C. Neutrophil extracellular traps kill bacteria. Science. (2004) 303:1532–35. doi: 10.1126/science.1092385 15001782

[91] Papayannopoulos V Metzler KD Hakkim A Zychlinsky A. Neutrophil elastase and myeloperoxidase regulate the formation of neutrophil extracellular traps. J Cell Biol. (2010) 191:677–91. doi: 10.1083/jcb.201006052 PMC3003309 20974816

[92] Zhang J Feng Y Shi D. NETosis of psoriasis: a critical step in amplifying the inflammatory response. Front Immunol. (2024) 15:1374934. doi: 10.3389/fimmu.2024.1374934 39148738 PMC11324545

[93] Ahrens S Zelenay S Sancho D. F-actin is an evolutionarily conserved damage-associated molecular pattern recognized by DNGR-1, a receptor for dead cells. Immunity. (2012) 36:635–45. doi: 10.1016/j.immuni.2012.03.008 22483800

[94] Del Fresno C Saz-Leal P Enamorado M Wculek SK Martínez-Cano S Blanco-Menéndez N. DNGR-1 in dendritic cells limits tissue damage by dampening neutrophil recruitment. Science. (2018) 362:351–6. doi: 10.1126/science.aan8423 30337411

[95] Cueto FJ Del Fresno C Sancho D. DNGR-1, a dendritic cell-specific sensor of tissue damage that dually modulates immunity and inflammation. Front Immunol. (2020). doi: 10.3389/fimmu.2019.03146 PMC7018937 32117205

[96] Murphy RC Bowers RC Dickinson J Zemski-Berry K. Perspectives on the biosynthesis and metabolism of eicosanoids. In: Curtis-Prior P, editor. The Eicosanoids. John Wiley & Sons, Ltd. Southern Gate, Chichester, West Sussex, England (2004). p. 3–16. The Atrium.

[97] Tsai AG Johnson PC Intaglietta M. Oxygen gradients in the microcirculation. Physiol Rev. (2003) 83:933–63. doi: 10.1152/physrev.00034.2002 12843412

[98] Bagher P Segal SS. Regulation of blood flow in the microcirculation: role of conducted vasodilation. Acta Physiol (Oxf). (2011) 202:271–84. doi: 10.1111/j.1748-1716.2010.02244.x PMC3115483 21199397

[99] de Wit C Griffith TM. Connexins and gap junctions in the EDHF phenomenon and conducted vasomotor responses. Pflugers Arch. (2010) 459:897–914. doi: 10.1007/s00424-010-0830-4 20379740

[100] Tallini YN Brekke JF Shui B Doran R Hwang SM Nakai J. Propagated endothelial Ca^2+^ waves and arteriolar dilation. vivo. Circ Res. (2007) 101:1300–9. doi: 10.1161/CIRCRESAHA.107.149484 17932328

[101] Knowles HJ Athanasou NA. Acute hypoxia and osteoclast activity: a balance between enhanced resorption and increased apoptosis. J Pathol. (2009) 218:256–64. doi: 10.1002/path.2534 19291710

[102] Arnett TR. Acidosis, hypoxia and bone. Arch Biochem Biophys. (2010) 503:103–9. doi: 10.1016/j.abb.2010.07.021 20655868

[103] Yao Y Cai X Ren F Ye Y Wang F Zheng C. The macrophage-osteoclast axis in osteoimmunity and osteo-related diseases. Front Immunol. (2021) 12:664871. doi: 10.3389/fimmu.2021.664871 33868316 PMC8044404

[104] Lewis JS Lee JA Underwood JC Harris AL Lewis CE. Macrophage responses to hypoxia: relevance to disease mechanisms. J Leukoc Biol. (1999) 66:889–900. doi: 10.1002/jlb.66.6.889 10614769

[105] Cramer T Yamanishi Y Clausen BE Förster I Pawlinski R Mackman N. HIF-1α is essential for myeloid cell-mediated inflammation. Cell. (2003) 112:645–57. doi: 10.1016/s0092-8674(03)00154-5 PMC4480774 12628185

[106] Tazzyman S Murdoch C Yeomans J Harrison J Muthana M. Macrophage-mediated response to hypoxia in disease. Hypoxia (Auckl). (2014) 2:185–96. doi: 10.2147/HP.S49717 PMC5045066 27774476

[107] Pfitzer G. Glatte muskulatur. In: Brandes R Lang F Schmidt R, editors. Physiologie des Menschen, 32nd ed. Springer-Lehrbuch, Springer-Verlag GmbH Deutschland, Heidelberg. (2019) p. 149–61. doi: 10.1007/978-3-662-56468-4_14

[108] Segal SS. Integration and modulation of intercellular signaling underlying blood flow control. J Vasc Res. (2015) 52:136–57. doi: 10.1159/000439112 PMC4670584 26368324

[109] Eto M. Regulation of cellular protein phosphatase-1 (PP1) by phosphorylation of the CPI-17 family, C-kinase-activated PP1 inhibitors. J Biol Chem. (2009) 284:35273–7. doi: 10.1074/jbc.R109.059972 PMC2790955 19846560

[110] Moczydlowski EG. Cellular physiology of skeletal, cardiac, and smooth muscle. In: Boron WF Boulpaep EL, editors. Medical Physiology, 3rd ed. Elsevier, Philadelphia, Pa. (2017) p. 228–51.

[111] Fisher SA. Vascular smooth muscle phenotypic diversity and function. Physiol Genomics. (2010) 42A:169–87. doi: 10.1152/physiolgenomics.00111.2010 PMC3008361 20736412

[112] Francis SH Busch JL Corbin JD Sibley D. cGMP-dependent protein kinases and cGMP phosphodiesterases in nitric oxide and cGMP action. Pharmacol Rev. (2010) 62:525–63. doi: 10.1124/pr.110.002907 PMC2964902 20716671

[113] Lüllmann-Rauch R Asan E. Glatte muskulatur. In: Lüllmann-Rauch R Asan E, editors. Histologie, 6th completely renewed edition. Georg Thieme Verlag, Stuttgart/New York (2019). p. 291–7.

[114] Parekh AB. Store-operated CRAC channels: function in health and disease. Nat Rev Drug Discov. (2010) 9:399–410. doi: 10.1038/nrd3136 20395953

